# Concerns of earthquake survivor mothers for their children and the role of school leadership in addressing them

**DOI:** 10.3389/fpubh.2025.1555125

**Published:** 2025-08-13

**Authors:** Meral Taner Derman, Şeyma Türen

**Affiliations:** ^1^Faculty of Education, Bursa Uludağ University, Bursa, Türkiye; ^2^Faculty of Education, Istanbul Okan University, Istanbul, Türkiye

**Keywords:** earthquake, concern, earthquake survivor mothers, child well-being, school leadership, post-disaster support, mothers’ concerns

## Abstract

**Introduction:**

This study aims to examine the change in the concerns of mothers with preschool children before and after the earthquake and the role of school administrators in relieving these concerns. Understanding these concerns is essential for improving post-disaster support strategies for families.

**Methods:**

The research was carried out in a holistic multi-case study design, which is one of the qualitative methods, with a study group consisting of 20 mothers and 5 school administrators living in the temporary housing area in Hatay. The data were collected through semi-structured interview forms and analyzed by content analysis method.

**Results:**

The findings showed that games and entertainment activities were at the forefront of mother–child relationships, but they were also a reflection of the earthquake. It has been revealed that mothers’ future plans focused on positive goals such as their children growing up in a healthy environment before the earthquake and becoming successful individuals by receiving a good education, but after the earthquake, these plans were negatively affected due to financial and social losses, uncertainties and disruptions in education. It has been shown that mothers had general concerns about the education, security and social development of their children before the earthquake, but these concerns became more concrete and intense after the earthquake. Mothers reported serious concerns, particularly about living conditions, psychological effects, education, and the future. School administrators, on the other hand, resorted to methods such as summer schools, social activities, guidance services and financial support to alleviate the concerns of mothers. They suggested improving housing conditions, opening vocational courses, strengthening infrastructure and involving psychosocial support teams.

**Conclusion:**

The research emphasized that the physical, social and psychological effects of natural disasters on individuals are multidimensional and revealed the critical role of school administrators in crisis management. It also highlights the need for coordinated efforts between families and schools in navigating post-disaster challenges.

## 1 Introduction

Natural disasters can be defined as natural events that are unpredictable, the area and level of impact they will affect cannot be predicted, and they cause devastating consequences for the surrounding living things and property losses (1, 2). These events turn into high-level disasters, straining local communities’ coping resources, often leading to serious social, societal, and economic losses (3, 4). Disasters have consequences such as extensive damage, economic hardship, or loss of life in one or more layers of society. While it usually occurs suddenly, it causes great harm and can take years to recover from its effects (5).

Earthquakes, which are among the most significant natural disasters, occur in most cases due to tectonic movements. These movements cause fractures in the Earth’s crust, producing seismic waves that radiate in all directions and shake the ground with varying intensity and magnitude. Occurring mainly along plate boundaries, tectonic earthquakes account for the vast majority of seismic events worldwide (6–9). Earthquakes, which are directly based on natural events and occur naturally, have been one of the most destructive types of disasters encountered throughout history (10, 11). Earthquakes seriously affect human life in a significant part of the world (12). According to evaluations of earthquake-related fatalities from the 20th century to the present, some of the countries most frequently affected by devastating earthquakes include China, Japan, Italy, Iran, Türkiye, Chile, Indonesia, and countries located along the Himalayan belt (13). Since Türkiye is located in an active earthquake zone, it is seriously affected by these disasters. Türkiye’s high seismic risk is largely due to its location along the Alpine-Himalayan seismic belt, one of the most active earthquake zones in the world. Moreover, the presence of several active tectonic structures—such as the North Anatolian Fault System (NAFS), East Anatolian Fault System (EAFS), Aegean Graben System (AGS), and the Bitlis Suture Zone (BSZ)—further increases the likelihood and intensity of earthquakes throughout the region (14–16). In Türkiye, as a final instance, on February 6, 2023, two high-intensity earthquakes with a magnitude (Mw) of 7.7 and 7.6 centered in Kahramanmaraş caused severe destruction, heavy damage and loss of life in 11 provinces (17, 18).

The social, economic, physical, psychological, and economic effects of natural disasters such as earthquakes occur in various dimensions (19–21). This disrupts the order of life, causing pain and an overwhelming sense of helplessness and hopelessness (22). The effects of disasters are felt more intensely, especially on children and vulnerable groups. Direct exposure to disasters is extremely stressful life events. Post-disaster stress, grief, and loss can lead to serious mental health problems in individuals, such as post-traumatic stress disorder (PTSD), and cause multiple developmental risks (20, 23–25). In this process, various psychological symptoms in individuals may occur at different levels (26). Grief, which is among the most common psychological reactions after disasters, is generally considered as a natural and almost universal response of individuals to extraordinary situations (3, 27).

Concern, an emotion that is often overlooked in the grief process, is recognized as a natural element of grief. Concern arises from the subjective thoughts of individuals and is defined as an ambiguous emotion. Concern, which has a great impact on human life, can shape the behavior of individuals (28, 29). From a psychological perspective, concern is conceptualized as a complex emotional response to uncertainty, perceived threat, or loss of control. It typically encompasses both cognitive and emotional elements and is frequently associated with negative anticipations about the future (30). This emotional state can impair essential cognitive functions such as attention, memory, and decision-making, thereby interfering with daily functioning (31). Furthermore, concern is influenced by broader social and cultural contexts; during periods of societal uncertainty or crisis, it tends to become more widespread and affects how individuals perceive and respond to their environment (32, 33). Natural disasters such as earthquakes can threaten psychological well-being in many ways and lead to both short- and long-term psychological distress, creating a significant mental health burden on affected individuals and communities. Post-earthquake concern, on the other hand, is a psychological reaction caused by traumatic events experienced during or after an earthquake. This concern arises due to factors such as the severity of the event, the duration of exposure and the individual’s perception of the event. It may manifest through cognitive, emotional, and physiological responses, and if left unaddressed, it can become chronic and impair daily life (34, 35). The impact of such concerns on the family structure is also noteworthy.

Families, as the primary caregiver of children, face great challenges during and after disasters. The deteriorating social structure after the disaster seriously prevents families from carrying out their regular activities and causes the family routine to change (23). It is stated that women are more affected by the earthquake than other individuals in this process, and the risk of developing psychological distress and depressive symptoms is higher than men (27, 36–40). Mothers, in particular, are more affected by their increased responsibilities and roles during this period, and experience more mental health questions than fathers. The earthquake affects women’s motherhood roles and exposes them to additional challenges such as taking care of family members and supporting their spouses. However, changes in parenting strategies can be observed as a result of natural disasters (25, 41–43, 73).

Effective interventions against the negative effects of disasters have a wide range of effects from individuals to societies (3, 44). Leaders play a critical role in this process. Leaders can support individuals and communities to reduce post-disaster distress by playing a supportive and constructive role in their coping with disasters (45). By taking on this leadership role, school administrators can make an important contribution to restoring order in the school environment after the disaster and creating a supportive school culture (46). Disaster preparedness increases the capacity of societies to cope with cold events. While school and community environments increase resilience against disasters, they contribute positively to the recovery processes of children and families (47, 48). For this reason, schools and school administrators should regularly update their pre-disaster awareness and post-disaster mitigation activities (49).

In conclusion, the effects of natural disasters on individuals and society are complex and multidimensional. Although the fact that natural disasters such as earthquakes are both common and highly worrying has brought the focus of attention to researching their effects on mental health, mental health problems and interventions related to them continue to be important (50, 51). In the literature, it is seen that studies on the effects of disasters on the mental state of mothers with preschool children are limited. Determining the social, socioeconomic and health consequences of the earthquake on women (41); examining the effects of adolescent children’s involvement in a disaster on mothers (52); revealing the impact of experiencing a natural disaster by the age of five on mental health and substance use disorders in adulthood (43); investigating family context and young children’s responses to earthquakes (53); About 3 years after the August 1999 earthquake in Türkiye, we identified the prevalence of post-traumatic stress disorder (PTSD) and concomitant depression (37); After the 2011 Van earthquake, studies examining the experiences of Turkish mothers living in the temporary housing area (54) were conducted. In addition to these, Arslan (55) examined the problems faced by school principals in schools after the February 6 Kahramanmaraş earthquake and the solutions to these problems. It is seen that research on the concerns of mothers of preschool children is insufficient. For this reason, in this study, it is aimed to determine the concerns of mothers who have preschool children and who directly experience the earthquake before and after the earthquake and to reveal the solution suggestions of school administrators who are considered as leaders in eliminating these concerns. In particular, the unique aspect of this study is to determine the solution suggestions of the leadership roles of school administrators in reducing the concern expressed by mothers in such traumatic situations. This study contributes by expanding the understanding of how parental concerns—particularly those of mothers with preschool children—interact with school leadership roles in post-disaster contexts.

## 2 Method

### 2.1 Research model

This research was carried out in the holistic multi-case study design, which is one of the qualitative research designs. In case studies, one or more individuals, facts, events or situations that the researcher cannot control, and the variables that affect or are affected by this situation, are handled within their own real-life conditions (56). In addition, case studies are a type of research that seeks answers to questions about a current situation and provides the researcher with the opportunity to collect in-depth data (57). In this study, this pattern was preferred because it was tried to determine the concerns of mothers with preschool children about their children before / after the earthquake and to determine what the school administrators, who are considered as leaders in eliminating these concerns, proposed as a solution. In this direction, firstly, the concerns of the mothers were determined and the solution suggestions of the school administrators were tried to be obtained with the interview form prepared for these concerns.

### 2.2 Study group

The study group was selected using criterion sampling, one of the purposive sampling methods. The inclusion criteria for the mothers were as follows: having a preschool-aged child; residing in Hatay, one of the provinces most severely affected by the earthquake; and currently living in temporary housing established in the region due to the destruction or severe structural damage of their home. 20 mothers who met these criteria and volunteered to participate in the study were included in the study group. The criterion for the selection of school leaders was that they were working as administrators in preschools located in the earthquake-affected region. 5 school administrators who met these criteria and volunteered to participate in the study were included in the study.

### 2.3 Data collection tools and data collection process

In the study, data were collected through interviews. Semi-structured interview forms were prepared by the researchers. Interview forms; It was sent to experts consisting of psychologists, psychological counselors and faculty members working in the field of preschool education. In line with expert opinions, the semi-structured interview form has been finalized. In semi-structured form prepared for interviews with mothers; 6 questions on demographic characteristics; there were 8 questions about their concerns about children. In the form prepared for interviews with school administrators, 9 questions for demographic information; there are eight questions to determine the solutions for what can be done to address the concerns of mothers.

In-depth interviews were conducted with mothers and school administrators who signed the voluntary participation consent form through semi-structured interview forms. The interviews were conducted individually in an empty classroom of a school in the temporary housing area where the mothers lived, and with the administrators in their rooms in their own schools. Before starting the interviews, the researchers introduced themselves and gave a brief explanation about the purpose of the study. Participants were informed that no personal or institutional information would be shared. Particular attention was paid to maintaining participant confidentiality, the research was carried out on a voluntary basis, and written consent was obtained from all participants. The study was conducted in accordance with relevant regulations and ethical guidelines. To build rapport, informal dialogue was initiated before the interview began. With participants’ consent, all interviews were audio-recorded using a digital recorder. The recordings were later transcribed verbatim and anonymized.

The interviews with the mothers lasted between 20 and 30 min, and the interviews with the managers lasted between 15 and 20 min. Throughout the interviews, the researchers adopted a neutral and empathetic stance, encouraging open communication while maintaining ethical sensitivity. In order to deepen the interviews, questions such as “What do you mean by this” and “Can you give an example?” Were also asked from time to time. Data saturation was observed when similar themes began to recur and responses from new participants no longer contributed meaningful new content to the study. At this point, the researchers decided to conclude the data collection process.

### 2.4 Analysis of data

The data of the study were analyzed by content analysis. The audio recordings taken during the interviews with a total of 20 mothers and 5 school administrators were transcribed by the researchers and converted into interview texts. The texts were analyzed by content analysis, and the codes and themes were determined separately by the two researchers. For the reliability analysis between encoders, the Miles & Huberman coefficient was calculated and determined as 89%. Based on the codes, the findings were explained under four themes related to the concerns of mothers and 7 themes related to the solutions of the concerns of school administrators.

### 2.5 Measures regarding the validity and reliability of the research

In order to increase the credibility of the research, expert opinion was consulted while preparing a semi-structured interview form. In order to enable the participants to respond sincerely, the purpose of the research was explained and permission was requested for audio recording. The identity information of the participants was kept confidential and codes were given to the participants. In order to increase the transferability of the research, the research process has been tried to be explained in detail. In order to increase the consistency of the research, direct quotations were made from the statements of the participants in the presentation of the findings. The researchers made separate codings and the percentage of compliance was calculated. In order to increase the verifiability of the research, detailed descriptions were made, the findings were written in detail, and the codes for the participants were arranged in a way that was appropriate to check the consistency of the data in the findings.

## 3 Results

As a result of the interviews conducted in this section, the findings were explained under four themes related to the concerns of mothers and seven themes related to the solutions of the concerns of school administrators.

### 3.1 Findings on mothers’ concerns

The findings regarding the mothers’ concerns were examined in four themes: “Mothers’ Relations with Their Children,” “Mothers’ Future Plans for Their Children and the Effect of the Earthquake on Plans,” “Mothers’ Concerns About Their Children Before the Earthquake and the Effect of the Earthquake on Their Concerns” and “Mothers’ Actions and Opinions on Eliminating Post-Earthquake Concerns.”

#### 3.1.1 Theme 1: mothers’ relationships with their children

The findings show that the most frequently emphasized theme in mother–child relationships is play and recreational activities (*f* = 21); in particular, gaming (*f* = 15) was frequently emphasized in this relationship. In addition, activities such as watching cartoons, painting and singing are also included in this category. While helping the lesson, reading books and doing activities at home play an important role in education and learning activities (*f* = 7), it is seen that children’s movement and exploration needs are met with outdoor activities (*f* = 6). While establishing good communication and developing a friendly relationship draw attention in the theme of emotional and social communication (*f* = 8), it is understood that spending time with household and kitchen chores in daily life and routine activities (*f* = 4) contributes to bonding. Finally, the category of post-earthquake special situations (*f* = 5) emphasizes the importance of post-crisis attachment and support efforts. Some of the answers given by mothers on this subject are as follows:

M3: *“When my daughter has a problem, I explain it to her in a realistic and proper way, not to distract her… We sing songs and play games together.”*

M6: *“I try to do the things he likes, we try to dispel this perception that he was usually affected by the earthquake.”*

*M14: “I do everything with my child. For example, I play games when necessary, I become a mother, I become a friend, I become a housewife. That’s how I communicate with them. I became more attached to my children after the earthquake…”*

#### 3.1.2 Theme 2: future plans of mothers for their children and the effect of the earthquake on plans

In the future plans of mothers for their children before the earthquake, the themes of living conditions (*f* = 9) and education life (*f* = 8) are mostly reached. While goals such as planning a good future in living conditions, living a healthy life and providing an environment where they will feel happy come to the fore, expectations for education life focus on children getting a good education and experiencing university life and growing up as successful and knowledgeable individuals. Plans to have a profession (*f* = 8) are associated with children getting good jobs and doing jobs they love. Religious and moral approach (*f* = 5) includes the goals of raising good children, providing religious education and gaining moral values. Within the scope of the uncertainty theme (*f* = 4), the inability to make a clear plan for the future and concerns about education come to the fore, and this situation is seen as one of the factors affecting the plans of some mothers. In general, mothers’ plans focus on ensuring that their children receive a good education, have a profession, lead a happy life and grow up with moral values. Some of the mothers’ plans for their children before the earthquake are as follows:

M2: *“I want my children to study in the future… I want them to live university life.”*

M5: *“… to have a better place to live.”*

M15: *“I want him to grow up well, I want him to study and have a profession with his own efforts.”*

In addition, it shows that mothers’ future plans for their children were deeply affected by the earthquake. The most frequently emphasized category in the future plans of mothers after the earthquake was material, moral and social losses (*f* = 9), and it was emphasized that expressions such as homelessness, disruption of the established order and the end of social life affected the plans. Influencing Education and Future Plans (*f* = 8) also has an important place; Factors such as disruption of school and education processes and disruption of kindergarten/nursery plans affect mothers’ concerns and plans. Psychological and Traumatic Effects (*f* = 5) manifest themselves with negative situations such as trauma, constant remembrance and fear. In the Concern and Uncertainty (*f* = 7) category, it stands out with situations such as affecting dreams and not being able to plan for the future. On the other hand, for only one mother, the earthquake had an accelerating effect on her ability to implement her plans (*f* = 1), which represents situations with minimal or no negative impact. In general, it is understood that the earthquake affected the future plans of mothers for their children in a multifaceted way and created uncertainty in many areas. Some of the maternal views on the theme are as follows:

M8: *“It did not have much of an impact for me. My thought before the earthquake was to move. Now we want to go there, and our family is not harmed. We will be a little faster in terms of moving.”*

M10: *“It had a very negative impact, I prepared everything a month in advance, but I cannot even visit all the programs right now. Because it affected us psychologically.”*

M18*: “The earthquake had a lot of impact. She could not go to the kindergarten I wanted, I could not give her the life I wanted, for example, I could not build her room right now.”*

#### 3.1.3 Theme 3: mothers’ concerns about their children before the earthquake and the effect of the earthquake on concern

**Figure 1:**
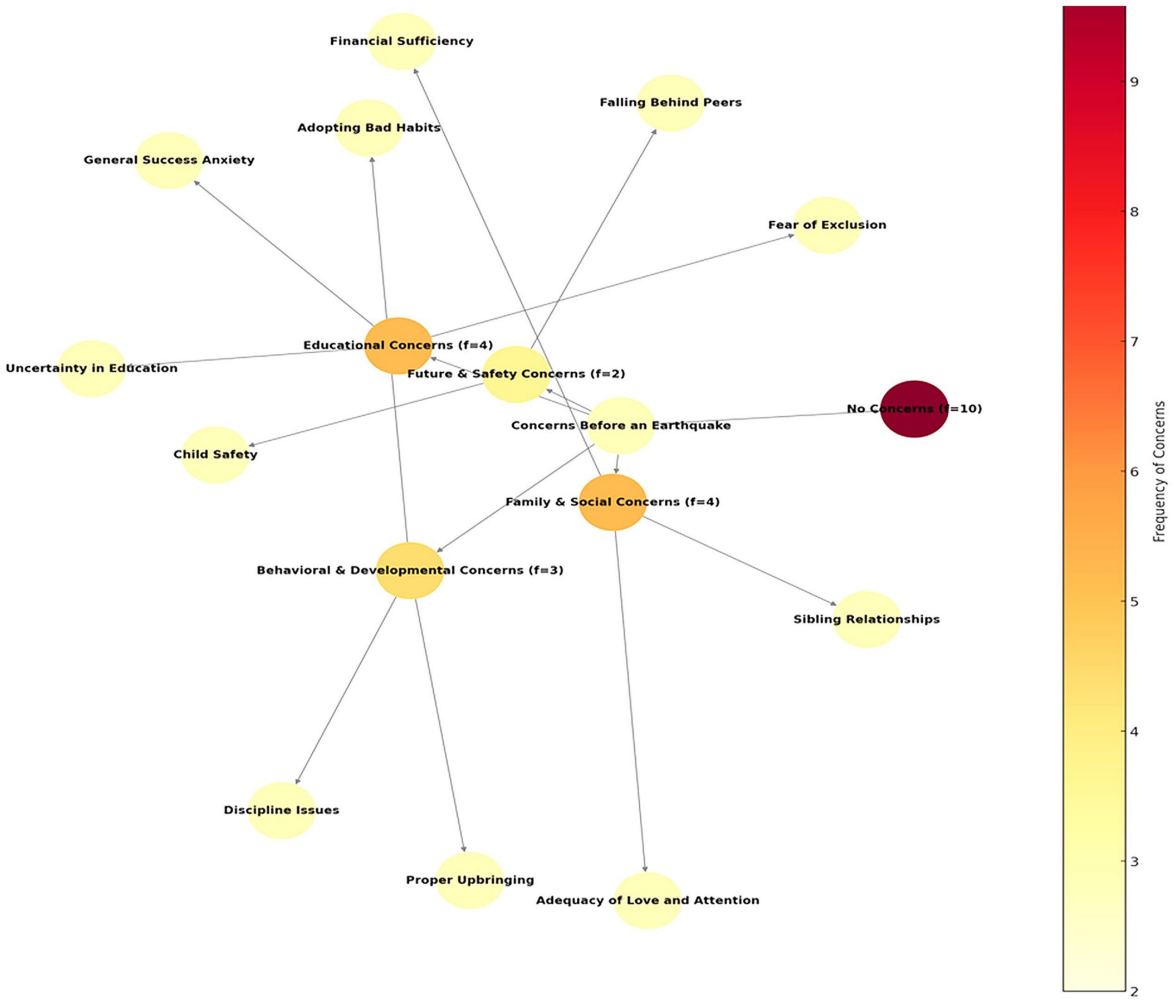
Mothers’ concerns about their children before the earthquake.

**Figure 2:**
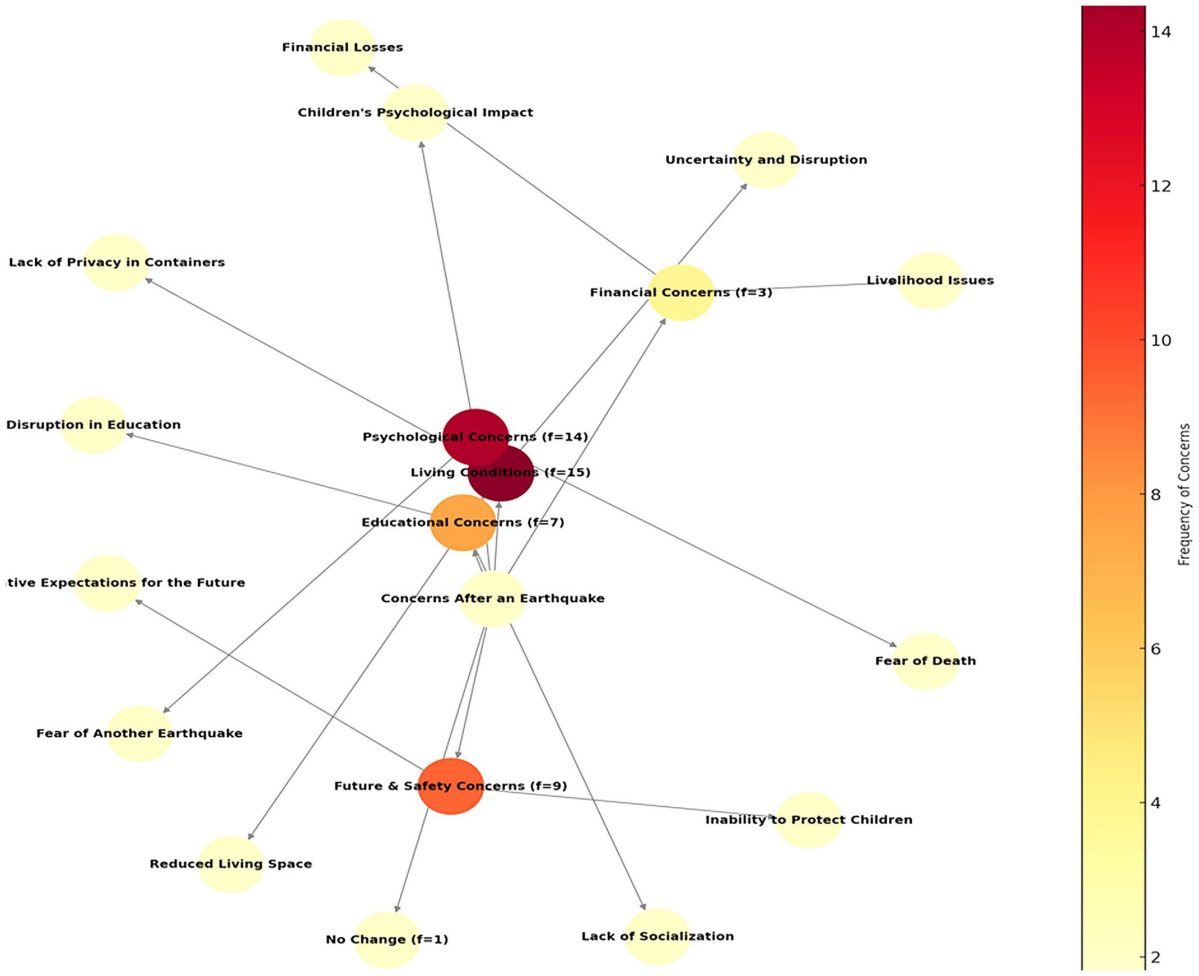
Mothers’ concerns about their children after the earthquake.

**Figure 3:**
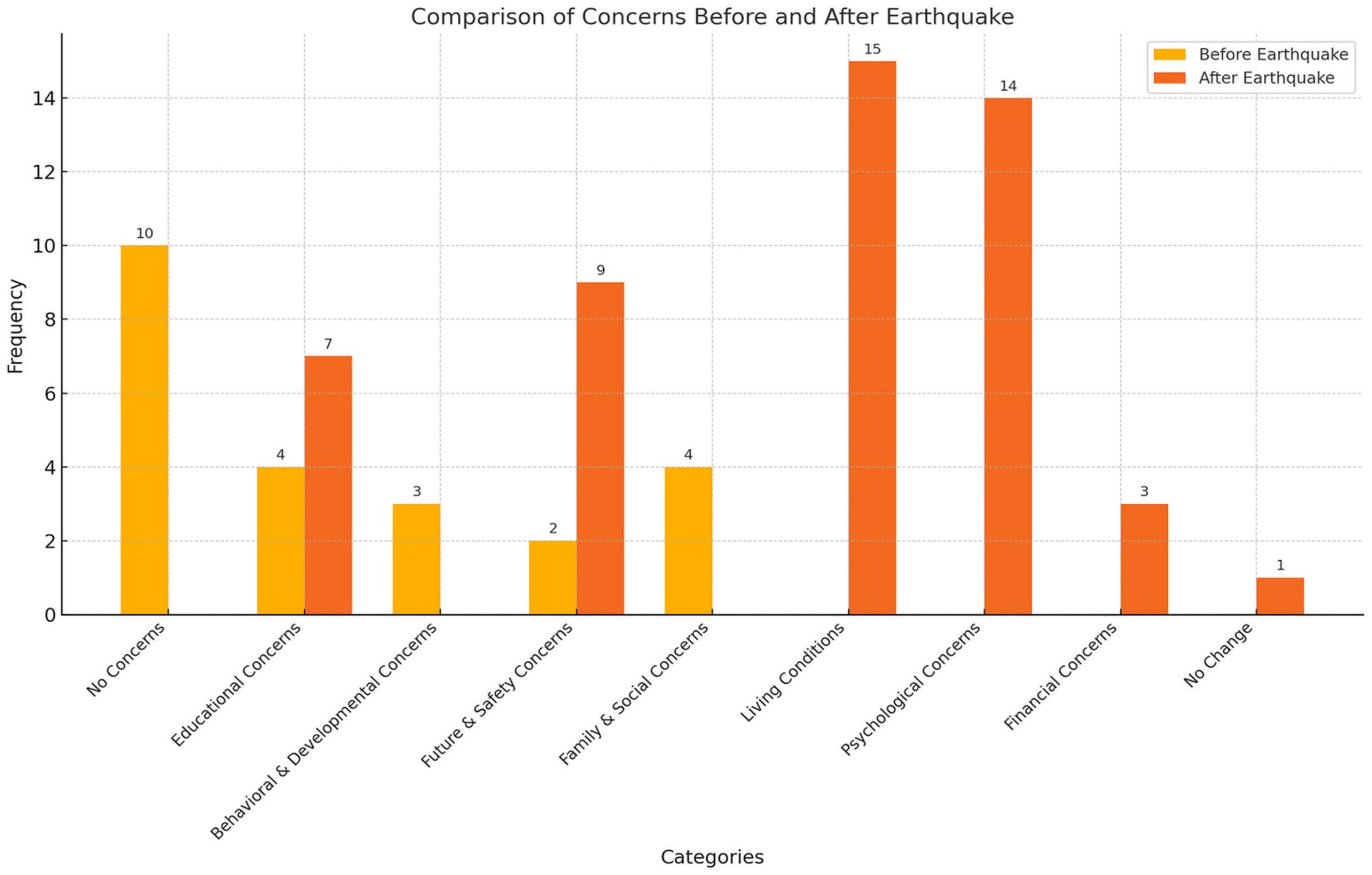
Change in mothers’ concerns about their children before and after the earthquake.

The category of No Concern (*f* = 10) came to the fore in the mothers’ concerns about their children before the earthquake, and it was observed that most mothers did not have any serious concerns about their children. In addition, Educational Concerns (*f* = 4) reflect uncertainties in children’s educational life, fear of exclusion and general success concerns. Behavioral and Developmental Concerns (*f* = 3) include sensitivities related to children’s discipline problems, acquiring bad habits and raising them as desired. General Concerns about Future and Security (*f* = 2) include the safety of children and fear of falling behind their peers, while Familial and Social Concerns (f = 4) include the adequacy of love and care in the family, relationships between siblings and the adequacy of financial means. Overall, it is understood that mothers’ concern is largely low, but there are certain sensitivities regarding education, security and family dynamics (Figures 1–3). Some of the maternal views on the theme are as follows:

M1: *“I did not have any concerns. I thought they would all have a good future, they would read.”*

M6: *“…. I was wondering if I could give him the attention he expected, if he would be the kind of kid I wanted, if he would do what I said.”*

M11: *“There were family problems among the children…”*

Mothers’ post-earthquake concerns are especially focused around living conditions (*f* = 15) and psychological concerns (*f* = 14). Problems such as the shrinkage of living spaces, the lack of privacy and uncertainty caused by staying in containers, and the deterioration of the living order were among the most frequently mentioned issues. Psychologically, deepening concerns such as fear of death, worries about earthquakes again, and psychological effects on children come to the fore. It is manifested by future and safety concerns (*f* = 9), fear of not being able to protect children, and negative expectations about the future. Education-related concerns (*f* = 7) focused on the disruption of children’s education and lack of socialization. Financial losses and livelihood problems (*f* = 3) are a significant concern, although they are relatively less mentioned. No change (*f* = 1) is a rarely expressed situation. In general, mothers’ concerns are predominantly shaped by living conditions and psychological effects. Some of the mothers’ post-earthquake concern expressions are as follows:

M4: “*No one knows how long it will take for the city to recover. I’m worried about schools. The class sizes are too large and not healthy. I’m very concerned about that*.”

M7: “*I do not know. Because we do not know if we will live in 1 h. Death is imminent at any moment, we cannot think about tomorrow.”*

M17: “*The fear of losing them for a moment … I’m so scared that we happen again and I cannot protect them*.”

When the change in mothers’ concerns about their children before and after the earthquake is examined, it is seen that the concerns become more intense and diverse after the earthquake. Psychological and physical effects have increased significantly compared to before the earthquake, and living conditions and psychological effects have become the main concerns. Financial concerns and livelihood problems have come to the fore as a new area of concern. After the earthquake, concerns about education increased, and new elements such as lack of socialization were added. Psychological concern, which was low before the earthquake, was one of the highest concern categories after the earthquake.

#### 3.1.4 Theme 4: mothers’ actions and opinions on relieving post-earthquake concern

When mothers’ methods of coping with their post-earthquake concerns are examined, the most common are Spending Time with the Child and Emotional Support (*f* = 7), Family Support and Cooperation (*f* = 5) and Psychological Support and Professional Assistance (*f* = 5). In these categories, efforts to try to understand the child, participate in social activities and make people forget the memory of the earthquake came to the fore, while providing solidarity with the spouse and getting expert support drew attention as important methods. In addition, mothers stated that they tried to cope with the situation within the framework of Trying to Be Positive and Strong (*f* = 5) and made statements about the Continuity of Education and Social Life (*f* = 3). However, some mothers explain that they are not eager to do anything with the expressions of Uncertainty and Hopelessness (*f* = 3). These data show that mothers mainly focus on supporting their children and try to manage this process with different strategies. Some of the mothers’ expressions in relieving concern after the earthquake are as follows:

M9: *“I do not know anything. I do not know anything about what’s going to happen in the future.”*

M12: *“I thought of it in one way, and I’ve already implemented it. I underwent psychological treatment for months … We’re fighting with his dad.”*

M19: *“I try to forget those moments. I make statements to my children and myself that it will not be repeated.”*

In addition to the steps taken by mothers to eliminate their concern, the most common opinions on how to relieve their concern are Education and Environmental Improvement (*f* = 7) and Psychological and Emotional Support (*f* = 7). Mothers find it important to get support from experts by improving the school environment, reorganizing the environment and strengthening themselves psychologically in reducing their concern. Within the scope of Family and Social Support (*f* = 3), methods such as participating in social environments, reassuring children and providing financial and moral support draw attention. Within the framework of Normalization and Continuation of Routine (*f* = 3), there are plans to return to the old order and a focus on positive memories. However, some mothers stated that they did not have a clear idea of the future with Uncertainty and Hopelessness (*f* = 3), while a small group expressed hope that their concern would decrease with their children’s future profession with Long-Term Thoughts (*f* = 1). This situation shows that mothers try to relieve their concerns with both short-term solutions and long-term expectations. Here are some mom views on the theme:

M2: *“I think education might be the best thing for my kids right now… Education is our priority and our best investment in our children.”*

M13: “*I have no idea, I still have not been able to think.”*

M16: *“We ask for help from teachers, whoever is interested…”*

M20: *“… I will be very happy if my daughters have a profession.”*

### 3.2 Findings on the resolution of school administrators’ concerns

When you examine the concerns of mothers about their children after the earthquake, it is seen that these concerns focus on education, future and security, financial losses and livelihood problems, and psychological concerns. In this section, the practices and suggestions of school administrators in addressing the relevant concerns are thematized separately. In addition, what needs to be done in the Container City and in terms of country policies was discussed. Related findings are “Practices and Suggestions for Eliminating Mothers’ Concerns About Education,” “Practices and Suggestions for Eliminating Mothers’ Concerns About Future and Security,” “Useful Practices and Suggestions for Eliminating Mothers’ Concerns About Living Conditions,” “Useful Practices and Suggestions for Eliminating Mothers’ Concerns About Financial Losses and Livelihood Problems,” “Eliminating Mothers’ Psychological Concerns Useful Practices and Suggestions on the Subject,” “Measures to Be Taken in the Container City to Address Mothers’ Concerns” and “Policy Recommendations to Address Mothers’ Concerns at the National Level.”

#### 3.2.1 Theme 1: practices and suggestions for addressing mothers’ concerns about education

School administrators’ efforts to reduce mothers’ concerns about education have been shaped around several main themes. Within the scope of Support with Education and Activities (*f* = 6), which is the area where it focuses the most, practices such as opening summer schools, focusing on social and game-based activities, organizing sports courses and making the school environment suitable for students are at the forefront. Studies on School and Building Safety (*f* = 4) include conducting technical research on building safety, clearly communicating the no-damage status, and addressing school consolidation concerns. Guidance and psychological support (*f* = 2) includes training on fears and traumas through guidance teachers, and information activities on what to do during an earthquake. Finally, within the framework of family participation and cooperation (*f* = 3), allowing parents to be present in the school area, encouraging communication with experts, and organizing activities in parent-school cooperation come to the fore. Some of the statements of school administrators about their practices are as follows:

SA1: *“… Especially for our students who will begin first grade, we opened a summer school and implemented an educational program that includes key sections to address learning gaps…”*

SA2: *“We opened a summer school. We signed a protocol with the Youth and Sports Directorate and served approximately 200 preschool students in our school with 5 h of free gymnastics courses per week.”*

SA3: *“Technical researches on building security were carried out by our administration, and summer school was opened to students who were missing. During the semester, activities were held in cooperation with parents and schools.”*

In addition to the practices carried out by school administrators, it is seen that the theme of “Enriching the School and Educational Environment” (*f* = 5) comes to the fore as the strongest theme with suggestions in eliminating the concerns of mothers about education. This theme is a prominent theme made to support both the physical and social needs of children; It covers a wide range from increasing children’s play and entertainment areas to making classroom and guidance rooms more attractive. The stated Summer Term Planning (*f* = 1) proposes the use of schools for various courses in the summer term and offers a solution to support the educational process. However, Uncertainty and Missing Idea (*f* = 1) reveal that different and innovative solutions have not yet been developed other than the application. Here are some of the suggestions from school administrators:

SA4: *“The school environment needs to be arranged according to the students and the school garden needs to be made suitable for play.”*

SA5: *“Equipping the classrooms with new and uncommon toys, purchasing materials from different sports branches for the school and adding them to the education system, equipping the guidance room in a way that attracts the attention of the student.”*

#### 3.2.2 Theme 2: practices and suggestions for addressing mothers’ concerns about the future and safety

They stated that school administrators carried out practices such as sharing documents regarding the security of the school, introducing the building and explaining the renovations made within the scope of School Safety Ensuring and Communication (*f* = 3) in order to eliminate the future and safety concerns of the mothers. In addition, it was said that they organized meetings on brochure preparation, expert trainings and post-crisis measures within the scope of Information and Training Activities (*f* = 4). Some of the statements of school administrators about their practices are as follows:

SA3: *“In meetings and private discussions, measures that can be taken after the crisis were discussed.”*

SA4: *“Their children were provided with a safe environment, they were made to know that they were safe.”*

SA5: *“A tour of the school building explained the renovations that have been made.”*

In addition to the practices of school administrators, Environmental Regulations (*f* = 5) come to the fore in the suggestions to eliminate the concerns of mothers about the future and safety. Participants emphasized the importance of concrete steps such as the rapid removal of the remnants of the earthquake, the solution of housing problems and the elimination of frightening images. In addition, it was stated that environmental arrangements should be made in coordination with the headman and district national education. Within the scope of Psychosocial Support and Activities (*f* = 2), supportive activities such as the preparation of child and parent play workshops and the regular continuation of the training of guidance services were suggested. However, it was observed that some participants did not offer any other different and innovative solutions and experienced uncertainty (*f* = 1). Some of the suggestions of school administrators are given below:

SA1: *“In order to alleviate the concerns, the remnants of the earthquake should be removed from the city as soon as possible. The housing problem of parents and students should be solved. Playgrounds and social event planning should be emphasized.”*

SA2: “*Children’s and parents’ play workshops can be prepared.”*

#### 3.2.3 Theme 3: practices and suggestions for addressing mothers’ concerns about living conditions

Social and Cultural Activities (*f* = 6) were the most common methods used by school administrators to eliminate mothers’ concerns about their living conditions. With this theme, steps such as organizing children’s festivals, planning family participation activities and presenting different social environments in the temporary housing area are emphasized. Within the scope of Guidance and Institutional Supports (*f* = 2), it was considered important to direct parents to the relevant institutions and to benefit from guidance services. In addition, under the category of Training and Courses (*f* = 1), courses were organized to alleviate the difficulties of temporary housing area life. Some of the statements of school administrators about their practices are as follows:

SA2: *“We tried to provide* var*ious opportunities by providing full-time education to children in order to reduce parents’ concerns about living conditions after the earthquake.”*

SA3: *“Children’s festivals were held in the temporary housing area. In addition, parents were referred to Family and Social Policies officials for support.”*

SA4: *“In order to reduce the fear of earthquakes, activities such as spending time with peers and playing games were carried out in a different environment.”*

In addition to their practices, school administrators also focus on the implementation of preparations and rapid action plans to normalize the functioning of the city under the theme of Rapid Response and Environmental Regulations (*f* = 2), as well as suggestions to address mothers’ concerns about living conditions. In addition, within the scope of Guidance and Social Activities (*f* = 2), suggestions for more effective use of guidance services and social activity clubs come to the fore. However, under the heading of No Idea (*f* = 3), it is seen that some participants could not develop a different and innovative solution proposal in this regard. Here are some of the suggestions from school administrators:

SA1: *“It is important to make a faster action plan against the reality of the earthquake, which quickly changes the concerns and living conditions of parents and students, and to make the city ready for its normal functioning.”*

SA5: *“Active use of the social activities club at school…”*

#### 3.2.4 Theme 4: practices and suggestions for addressing mothers’ concerns about financial losses and livelihood problems

Financial Support (*f* = 6) and Coordination of Aids (*f* = 4) come to the fore in addressing the concerns of school administrators about mothers’ financial losses and livelihood problems. In this context, with the cooperation of the governorship and aid organizations, practices such as supplying materials, meeting student needs and distributing educational materials were carried out. Some of the practices of school administrators are as follows:

SA1: *“No demands were made from the students after the earthquake. In addition, we provided toys and materials for our students to do activities in tents or containers. Students who had financial difficulties were referred to the social assistance foundation. It served as a bridge in the activities and support of associations and foundations authorized by the governorship.”*

SA3: *“… Educational materials were distributed.”*

School administrators have made various suggestions to address mothers’ concerns about financial losses and livelihood problems. Within the scope of financial support and aids (*f* = 4), it was recommended to provide regular financial aid, to cover education-related transportation, stationery, activity and food expenses with allowances and to maintain existing supports. Economic arrangements and facilities (*f* = 3) include tax amnesty for tradesmen, civil servants and workers in the region, cancelation or proper restructuring of credit card and loan debts, and reduction of training costs. Here are some of the suggestions from school administrators:

SA2: *“Tax amnesties, appropriate deletion of credit card loan debts can be made for all kinds of occupational groups working in the earthquake zone, civil servants, workers.”*

SA5: *“… Continued support from benefactors and appropriations from the ministry…”*

#### 3.2.5 Theme 5: practices and suggestions for mothers’ concern about psychological concerns

The practices of school administrators in eliminating the concerns of mothers about their psychological concerns were grouped under a single Psychological Support and Information category (*f* = 5). Information and support activities such as guidance service support, preparation of family bulletins and organization of seminars were carried out to eliminate psychological concerns. Here are some of the suggestions from school administrators:

SA1: *“We prepared regular family bulletins and informed our parents about how to cope with the fear of earthquakes…”*

SA3: *“Parents who expressed concerns about the school and the student were supported by the guidance service.”*

SA4: *“The school guidance service has been working on the earthquake.”*

School administrators have made various suggestions to eliminate the psychological concerns of mothers. Under the category of Expert Interventions and Psychosocial Support (*f* = 3), it was recommended that mass support should be provided by specialist physicians in cities after aftershocks, that psychologists and psychiatrists should actively participate in the process, and that psychosocial support teams should regularly reach individuals affected by the earthquake. Within the scope of Guidance and Family Support Services (*f* = 3), it is important that family judges follow up with families, that the guidance service continues to work on earthquakes, and that regular meetings and seminars are held with parents. Lack of Ideas and Uncertainty (*f* = 1) did not offer a different and innovative solution. Here are some of the suggestions from school administrators:

SA2: *“It would be beneficial for the teams to reach the individuals affected by the earthquake living in the city on a regular basis, to carry out activities and to reach the people in a certain order, such as the family doctor following the family rather than having a one-time interview on paper.”*

SA5: *“Regular meetings and seminars between the guidance service and the parents …”*

#### 3.2.6 Theme 6: measures to be taken in the container city to address mothers’ concerns

Among the suggestions made by school administrators for addressing mothers’ concerns in the temporary housing area, Normalization and Support Studies (*f* = 6) were most frequently emphasized. These included steps such as restoring daily routines, providing scientific information, transitioning to prefabricated school buildings, and implementing regular follow-up mechanisms for families. Additionally, proposals related to Vocational Skills Acquisition and Contribution to Production (*f* = 2) highlighted the importance of offering vocational courses and involving residents in productive activities. Suggestions under the category of Social and Cultural Activities (*f* = 2) included expanding cultural events and establishing support offices to enhance community well-being. Here are some of the recommendations expressed by school administrators:

SA1: *“Public education center courses and vocational courses contribute to the citizens who are included in the production, both to relieve their future concerns and to contribute to the development of the citizens who are constantly expecting and getting used to comfort…”*

SA2: *“With the transition of container living spaces to prefabricated structures, at least people do not remind them of the earthquake, and normalization is one step closer.”*

SA5: *“An office can be established in each temporary housing area by the Ministry of Family and Social Policies, Religious Affairs, Red Crescent and close follow-up of families can be ensured…”*

#### 3.2.7 Theme 7: policy recommendations to address mothers’ concerns at the national level

School administrators offered several policy-level recommendations to address the concerns of mothers, focusing on key areas of disaster response and recovery. Under the theme of Crisis Management and Rapid Response (*f* = 2), participants emphasized the need to accelerate crisis response mechanisms and conduct debris removal activities simultaneously with post-disaster interventions. Within the scope of State Intervention and Effective Public Services (*f* = 3), it was stated that the presence of the state should be strongly felt through the provision of various services, that bureaucratic processes should be expedited, and that problems should be addressed on-site. Regarding Structural and Infrastructure Reinforcement (*f* = 2), it was suggested that post-earthquake building inspections should be conducted based on fault lines and that existing buildings should be strengthened accordingly. In relation to Financial Support and Incentives (*f* = 2), the continuation of both financial and psychological support and the increase of state-provided incentives were recommended. Finally, the theme of International Perspective (*f* = 1) emphasized the importance of examining how other countries have dealt with similar earthquake experiences. Here are some of the recommendations expressed by school administrators:

SA2: *“According to the fault lines on the earthquake maps, the building stocks can be reviewed again and the buildings can be strengthened.”*

SA3: *“To investigate the methods of countries that have experienced these events and have been successful in addressing the concerns.”*

SA4: “*The authorities need to provide realistic and scientific information… It is very important that the presence of the state is felt in a concrete way in order to address people’s concerns.”*

## 4 Discussion

In line with the findings obtained as a result of mothers’ definition of their relationships with their children, it has been determined that games and entertainment activities are at the forefront in mother–child relationships, as well as education, emotional communication, daily living activities and post-earthquake special situations. Some of the studies conducted in the literature are similar to our findings. In the study conducted by Yumbul et al., (54), it was determined that the earthquake and the resulting relocation caused various changes between mother–child relationships. Disasters such as earthquakes can cause differences in the roles of mothers (41). The nature of the parent–child relationship with the mental health of parents related to the earthquake is related to the negativities experienced by their children (53, 58). In addition to the fact that the earthquake affects the mother–child relationship, the highest rate is that games and entertainment activities are prioritized in their relationships. As a result of the studies conducted by Nawa et al. (59), it is stated that play-based mother–child relationship after natural disasters is preventive for child behavior problems. For this reason, it is thought that game-based interactions can contribute to alleviating the psychological effects of such traumas by ensuring that children are relatively less harmed by negative environmental effects such as earthquakes.

Before the earthquake, where mothers’ plans for the future of their children before and after the earthquake were examined, mothers focused on positive goals such as their children growing up in a healthy environment, becoming successful individuals by receiving a good education, gaining religious-moral values and having a profession, but these plans were negatively affected after the earthquake. Concerns about material, moral and social losses and disruption of education and future plans have come to the fore. Psychological traumas have changed the plans of mothers and uncertain the future plans of some mothers. With the impact of the earthquake, mothers’ expectations about their children’s future have changed drastically, causing them to reshape their plans to a great extent. The negative effects of the earthquake are not only limited to physical losses and deterioration in living conditions, but also lead to an increase in mental health problems and psychological traumas (60). However, these effects are not always long-lasting (61). In the research conducted by Güngör (62), while the disruption of education immediately after the earthquake was meaningless, it started to be seen as an important problem in the following days. However, in the same research, very similar to our findings, it is stated that there are individuals who say that there is no plan after the earthquake and that they cannot see ahead. This situation reveals the significant changes and uncertainties experienced in the plans of mothers about the future of their children after the earthquake, but also shows that mothers can enter into a transformation of setting new goals and restructuring during the recovery process.

When mothers’ concerns about their children before and after the earthquake were examined, a significant change emerged. Before the earthquake, the concerns were more general, low-intensity and focused on children’s educational achievement, behavioral development and family dynamics, but after the earthquake, these concerns emerged in a much more concrete, deepened way that directly affected daily life. Psychological and physical effects and concerns about education have increased significantly compared to the pre-earthquake period, as well as financial concerns, livelihood problems and lack of socialization have become new areas of concern. While physical problems such as deterioration of living conditions, inadequacy of shelter areas and lack of privacy came to the fore, psychological concerns such as fear of death and concern about experiencing an earthquake again were also expressed by mothers. In addition, there is concern about the inability to protect children and the disruption of their education, financial losses and security. Notably, approximately half of the mothers reported having no serious concerns about their children before the earthquake. This lack of pre-disaster concern may reflect a normalization of seismic risk, limited awareness of regional vulnerability, or culturally embedded fatalistic beliefs that reduce perceived control over disaster outcomes. The findings and similar studies in the literature show that disasters cause various concern states. Studies show that disasters have profound psychological, social and physical effects on individuals. It is emphasized that various psychological problems such as post-traumatic stress disorder (PTSD), depression, grief, panic disorder, concern and substance use disorders are common after disasters (8, 25, 63). In addition, it is stated that traumatic events such as earthquakes can cause grief, concern, stress-related health problems and suicidal thoughts in individuals, and these effects deepen the sense of insecurity (5, 23). In some, it is stated that they feel that the earthquake is happening again and again (62). However, the cramped, noisy and lack of safety measures in post-disaster shelter areas cause sleep problems and a decrease in the feeling of security (3, 41). When these findings and the data in the literature are evaluated together, it is seen that disasters are not limited to physical losses, but also affect various aspects such as mental health, daily life dynamics and future perception of families in a multidimensional way.

Mothers often resort to methods such as spending time with their children, providing emotional support, family solidarity, psychological help and trying to be positive in order to reduce their concern after the earthquake. In addition to these, mothers state that education and improvement of the environment, psychological and emotional support, social support, and return to the old order can be effective in alleviating their concern. Some mothers, on the other hand, are in a state of uncertainty and despair and cannot think of a clear solution. This situation reveals that mothers try to manage their concern with short-term solutions and long-term expectations. It is expected and natural to enter a mourning process immediately after the earthquake. At this point, it is considered important to treat grief if it does not become complicated. Different psychosocial interventions are carried out for psychological problems that are often observed in the form of traumatic stress, concern, and depressive symptoms (26, 63). Studies have shown that in addition to psychosocial support, resilience, social bonds and efforts to re-establish daily routines after adversity are important in recovery processes (64, 65). Based on the mothers’ statements, it can be said that both individual and social solidarity are critical in the face of challenges, and mothers strive to maintain their hope for the future.

In order to address the concerns of mothers about education, school administrators have mostly turned to activities such as organizing summer schools, social and game-based activities, and ensuring building security. In addition to these, it is seen that the physical and social enrichment of school and educational environments is the most emphasized solution among the suggestions to eliminate concerns about education. In order to address mothers’ concerns about the future and safety, school administrators focused on practices such as sharing documents related to school security, explaining building renovations and information meetings. In addition to these, the most frequently emphasized issue of school administrators’ suggestions regarding the future and security is the rapid implementation of environmental arrangements such as removing the remnants of the earthquake and solving housing problems. In addition, psychosocial support activities such as play workshops for children and parents should be carried out on a regular basis. When the literature is examined, it is seen that as a result of the research carried out by Arslan (55) with school administrators, very similar results were obtained to our findings. In the relevant research, school administrators conducted psychosocial studies for students, parents and teachers after the earthquake; it focused on extracurricular activities and carried out individual student follow-up to solve school attendance problems. Documents regarding the undamaged nature of the schools were shared, meetings were held with teachers and parents, and compensatory trainings were organized in order not to fall behind in education. The deterioration in children’s functioning after a disaster is becoming more evident, especially in schools, with damaged physical conditions, disrupted routines, and reduced social support (66). Schools are important centers not only for education, but also for increasing children’s psychosocial resilience. Research highlights that school environments strengthen children’s sense of security and provide social support (67). In this context, the efforts and recommendations of school administrators can be considered important not only for ensuring the continuity of the educational process but also for enhancing children’s psychosocial resilience and reinforcing families’ confidence in the future.

School administrators most frequently resorted to social and cultural activities to reduce mothers’ concerns about living conditions; it organized children’s festivals, encouraged family participation and provided social environments in the temporary housing area. In their recommendations, rapid intervention and environmental arrangements, as well as more effective use of guidance and social activities were emphasized in order to improve living conditions. School administrators have mostly focused on the coordination of financial support and assistance to address mothers’ concerns about financial losses and livelihood problems. Through the governor’s office and aid organizations, the supply of materials, meeting the needs of students and distributing educational materials were provided. In their proposals, it was emphasized to maintain regular financial assistance and to provide economic facilities. School administrators organized guidance service support, family bulletins and seminars and information activities to address the psychological concerns of mothers. In the literature, it is stated that it is considered important to carry out the practices implemented and recommended by school administrators after the earthquake. It is necessary to create a disaster management plan in order to protect children from disasters or to receive minimal damage (47). Parents rely on outside help to rebuild their lives and start normal life as a family unit. While receiving these aids inadequately or late leads to negativity, parents should see that their children are healthy and have an adequate level of social support and psychological help are among some factors that can protect their mental health (23, 25). In addition, disasters can be devastating not only psychologically or socially, but also in terms of the livelihoods of survivors. The collective effects of natural disasters spread across social and political spheres, so it is important to take precautions (68). In addition to all these, it is considered necessary to carry out awareness-raising and awareness activities as well as survival both before and after disasters (41). Moreover, local health centers can support mothers in regulating their negative emotions (54). In this context, it can also be stated that school administrators consider it significant, through their actions and suggestions, to improve families’ living conditions and support children’s development not only during times of crisis but also in the long term.

In order to alleviate the concerns of mothers in the temporary housing area, school administrators suggested steps for the normalization of life, scientific information, transition to prefabricated buildings and regular follow-up of families. In addition, proposals such as the opening of vocational courses and the participation of citizens in production have also come to the fore. Within the scope of social and cultural activities, it was emphasized that cultural activities should be increased and support offices should be established. School principals emphasized the importance of country policies, accelerating crisis management in order to address mothers’ concerns, a concrete presence of the state, accelerating bureaucratic processes and listening to problems on site. In addition, they suggested that within the scope of infrastructure strengthening, structures should be reviewed according to fault lines after the earthquake and buildings should be strengthened. It was also stated that financial support should be increased and the earthquake experiences of other countries should be investigated. It is stated in the literature that similar suggestions are important. It has been noted that the effects of displacement on mental health differ according to housing experiences (60). Research conducted by Ali et al., (69) showed that those living in temporary housing have higher stress levels, and that permanent and safe housing facilities play a critical role in reducing such problems. Meeting the shelter needs and improving the living conditions after the earthquake is considered as a priority step for the mental and physical well-being of the society. Close to the findings of this research, Güngör (62) stated that the biggest demands of individuals affected by the earthquake are to solve their housing problems, and pointed out that concerns can be alleviated with solutions such as providing houses. In the post-disaster period, it is of great importance to rapidly normalize life and meet the vital needs of the affected communities (70). In this process, it is important that rapid assessments are made by aid organizations and basic needs are met (71). In addition, it is emphasized that structural measures such as strengthening school buildings and residences should be taken in order to be prepared for disasters (72). All these suggestions and findings reveal the necessity of addressing both physical and psychosocial needs in a balanced way in the post-earthquake period. With effective crisis management and community-centered interventions, it will be possible not only to solve current problems, but also to increase resilience against future disasters.

## 5 Limitations and recommendations

This study is limited to 20 mothers living in Hatay and 5 school administrators working there. The importance of the direct impact of the earthquake can be understood more clearly by expanding the working group of the research and conducting similar studies with mothers and school administrators in the regions directly and indirectly affected by the earthquake. As qualitative research, it is limited to the findings obtained from the interview form used in the data collection method. By conducting new studies designed with a mixed method, it can be ensured that the findings obtained are both generalizable and detailed. Only school administrators working in schools were included in the study. By designing new research and involving school administrators, teachers and other public servants working in the school, concerns and suggestions can be addressed from other perspectives. In addition to all these, the concerns expressed by the mothers and the valuable suggestions of the school administrators can be made more widely and interventions can be carried out together with common stakeholders.

## Data Availability

The data analyzed in this study is subject to the following licenses/restrictions: the datasets generated during and/or analyzed during the current study are available from the corresponding author on reasonable request. Requests to access these datasets should be directed to Şeyma Türen, seymaturen@hotmail.com.
