# Supplementary material for: Concerns of earthquake survivor mothers for their children and the role of school leadership in addressing them
Source: Front Public Health. 2025 Aug 13;13:1555125. doi: 10.3389/fpubh.2025.1555125 (PMC12380865; doi:10.3389/fpubh.2025.1555125)
Supplement: Supplementary file 1 [file Supplementary_file_1.pdf]

frontiers

Concerns of Earthquake Survivor Mothers for Their Children and the Role of School Leadership in Addressing Them

Supplementary Material

1 Supplementary Figures and Tables

1.1 Supplementary Tables

Table 1. Themes, categories and codes used in the mothers' findings

Theme	Category	Code	*f*
**Mothers' Relationships with Their Children**	Games & Entertainment Activities	Playing games (M3, M4, M5, M6, M9, M11, M12, M13, M14, M15, M16,M17, M18, M19, M20)<br>Watching cartoons (M1, M6, M13)<br>Singing songs (M3)<br>Painting (M4, M16)	21
	Education and Learning Activities	Helping with class (M2)<br>Reading books together (M2)<br>Teaching to communicate (M3)<br>Doing activities at home (M5,M11A,16)<br>Playing educational phone games (M18)	7
	Outdoor Activities	Playing Outside (M5, M12)<br>Picnic (M16)<br>Cycling/scooter riding (M10)<br>Sightseeing (M12)<br>Going to the park (M16)	6
	Establishing Emotional and Social Communication	Communication based on love and respect (M2)<br>Communicate well (M3, M10, M11, M14, M15)<br>Explain problems realistically and properly (M3)<br>Trying to do what the child wants (M14)<br>Establishing a friendly relationship (M12)<br>Taking on different roles such as mother and friend (M14)	10

	Daily Life and Routine Activities		Helping the mother with household chores (M8, M10)<br>Spending time together with kitchen chores (M2, M12)	4
	Special Conditions for Earthquakes		Spending time outside the container (M2)<br>Trying to dissipate the perception of an earthquake (M6)<br>Experiencing the effects of being under the rubble (M7)<br>Connecting more after an earthquake (M14)<br>Supporting after an earthquake (M15)	5
**Future Plans of Mothers for Their Children and the Effect of the Earthquake on Plans**	Pre-Earthquake Plans	Education Life	Asking them to study in the future (M2, M9, M17)<br>Wanting them to live university life (M2, M16)<br>To ensure that they receive a better education (M5)<br>Wanting them to be educated and knowledgeable (M8)<br>Succeed (M8)	8
		Having a Profession	Want them to have good jobs (M1, M2, M3, M20)<br>Planning to study and have a profession (M15, M16, M19)<br>Thinking about doing what they love (M17)	8
		Living Conditions	Moving to a nice house (M3)<br>Planning a beautiful future (M3, M14, M19)<br>Providing a good living space (M5)<br>Be healthy (M18)<br>Plan to do whatever the child wants (M7)<br>Being where they feel happy (M17)<br>Wanting them to recover from the effects of the earthquake (M14)	9
		Religious and Moral Approach	To provide religious education (M11)<br>To ensure that they grow up as good children (M3, M8)<br>To have a good upbringing (M15)<br>To be respectful and loving (M8)	5
		Ambiguity	Worrying about not having a proper school (M4)<br>Not being able to think about the future (M6, M15)<br>Not planning for the future (M10)	4

	The Impact of the Earthquake on the Plans	Anxiety and Uncertainty	Uncertainty of the end (M1)<br>Inability to make future plans (M6, M20)<br>Worry that plans are difficult and time-consuming (M2, M3, M19)<br>Influence of dreams (M7)	7
		Material, Moral and Social Losses	Material losses (M11, M12, M17)<br>Moral losses (M11)<br>Disruption of the established order (M6, M17)<br>Homelessness (M5, M18)<br>End of social life and activities (M4)	9
		Psychological and Traumatic Effects	Experiencing trauma (M5)<br>Constant remembering and experiencing fear (M11, M13, M19)<br>Psychological effects (M10)	5
		Influencing Education and Future Plans	Affected school and education plans (M15, M16)<br>Disruption of kindergarten/nursery plans (M7, M18)<br>Very affected (M4, M7, M9, M13, M14)	9
		Minimal/No Negative Impact	Accelerated the realization of plans (M8)	1
**Mothers' Concerns About Their Children Before the Earthquake and the Effect of the Earthquake on Their Concerns**	Pre-Earthquake Concerns	No Anxiety	No anxiety at all (M1, M2, M4, M7, M9, M12, M14, M15, M18, M19)<br>Less anxiety than ever (M5)	11
		General Concerns About Education	What education will be like (M10)<br>Exclusion at school (M17)<br>General concerns about education (M5, M20)	4
		Behavior and Developmental Concerns	Not being able to raise the child as he wants (M6)<br>Fear of not being able to discipline (M11)<br>Fear of acquiring bad habits (M16)	3
		General Concerns About the Future and Security	Fear of falling behind peers (M8)<br>Concern for the safety and harm of the child (M13)	2
		Family and Social Concerns	Adequacy of the level of care and love in the family (M8)<br>Don't worry about not getting what you want (M13)<br>Family troubles (M11)<br>Conflicts between siblings (M17)	4

	The Effect of the Earthquake on Concerns	Growing Concerns About Education	Uncertainty of education (M1, M10, M16)<br>Concern about falling behind in terms of education (M2)<br>Not being able to create a good education area (M5)<br>Increasing class size (M4)<br>Inability of children to socialize (M2)	7
		Growing Concerns About the Future and Security	Fear of not being able to protect children (M1, M13, M17, M19)<br>Worry about safety (M10, M19)<br>Negativity in looking to the future (M6, M7, M12)	9
		Concerns About Living Conditions	Lack of children's study space and personal space (M3, M16)<br>Staying in a container (M5, M20)<br>Lack of privacy (M10, M20)<br>Crowdedness of the container city (M10, M11)<br>Disruption of life order (M11, M14, M15)<br>Uncertainty of returning to the way life was restored (M2, M4)<br>Fear of the changing environment (M1, M8)	15
		Financial Losses and Livelihood Problems	Material and moral difficulties (M12)<br>Everything is missing (M14)<br>Nothing left (M9)	3
		Psychological Concerns	Fear/anxiety of death (M7, M13)<br>Fear of earthquake/destruction again (M13, M14, M15)<br>Living with constant fear (M6)<br>Being psychologically affected (M11, M12)<br>Psychological effects of children and sleep problems (M11)<br>Deepening of existing anxieties (M3, M9, M16)<br>Fear of living in an apartment again (M6, M8)	14
		No Changes	Nothing has changed (M18)	1
		Family Support and Collaboration	Supporting the child with the spouse (M2)<br>Struggling with the spouse (M3, M12)<br>Chatting (M15, M16)	5
	What is done to eliminate concerns	Psychological Support and Professional Assistance	Consultation with a psychologist (M8)<br>Receiving psychological support (M10, M12, M17)<br>Talking to teachers and dealing with them (M11)	5

**Mothers' Actions and Opinions on Eliminating Post-Earthquake Anxiety**		Spending Time with the Child and Emotional Support	Keeping the child close (M1)<br>Spending more time with the child (M6)<br>Trying to understand him (M6)<br>Participating in social environments and supporting the child (M5, M8)<br>Trying to forget the moment of the earthquake (M13, M19)	7
		Don't Try to Be Positive and Strong	Stand even stronger (M2)<br>Trying to look at life positively (M2, M14)<br>Trying to manage the situation by behaving normally (M17)<br>Facing one's concerns directly (M18)	5
		Maintaining Education and Social Life	Fighting for the continuation of children's education (M4, M5)<br>Participating in social situations (M5)	3
		Uncertainty and Hopelessness	Lack of enthusiasm to do anything (M7, M9, M20)	3
		Family Support and Collaboration	Supporting the child with the spouse (M2)<br>Struggling with the spouse (M3, M12)<br>Chatting (M15, M16)	5
	Their Views on Relieving Concerns	Education and Environmental Improvement and Remediation	Believing that it will improve in the school environment/with education (M2, M5, M6, M12, M18)<br>Reconstruction of new schools (M4)<br>Arrangement of the environment (M6)	7
		Psychological and Emotional Support	First of all, psychological and emotional self-empowerment (M3)<br>Receiving psychological support (M10, M15, M19)<br>Getting help from experts (M8, M16)<br>Becoming aware (M11)	7
		Family and Social Support	Starting to participate in social situations (M10)<br>Reassuring children (M14)<br>Providing financial and moral support (M18)	3
		Normalization and Continuation of Routine	Planning to go home and go back to the old order (M1)<br>Don't try to pretend that the earthquake didn't happen (M17)<br>Remember the good times (M14)	3
		Long-Term Considerations	Believing that children will get better when they get a job (M20)	1

		Uncertainty and Hopelessness	Lack of an opinion yet (M7, M9, M13)	3
		Education and Environmental Improvement and Remediation	Believing that it will improve in the school environment/with education (M2, M5, M6, M12, M18)<br>Reconstruction of new schools (M4)<br>Arrangement of the environment (M6)	7

Table 2. Themes, categories and codes used in school administrators' findings

Theme	Sub-Theme	Category	Code	*f*
**Practices and Suggestions for Eliminating Mothers' Concerns About Education**	Applications in Relieving Concerns	Support with Training and Events	Emphasis on social activities and game-based education (SA1)<br>Opening a summer school for students (SA1, SA2, SA3)<br>Organizing free gymnastics courses by making a protocol with the Youth Sports Directorate (SA2)<br>Organizing the school environment according to the students (SA4)	6
		Studies for School and Building Security	Demonstration that the building is undamaged (SA1, SA5)<br>Technical research on building safety (SA3)<br>Explaining that there will be no merger with other schools (SA5)	4
		Guidance and Psychological Support	Providing training on fears and traumas by the guidance teacher (SA5)<br>Informing parents and students about what to do during an earthquake (SA1)	2
		Family Involvement and Collaboration	Allowing parents to wait in the classroom and school yard (SA1)<br>Parents' contact with expert counselors and teachers (SA1)<br>Organizing activities in cooperation with parents and schools during the term (SA3)	3
	Suggestions for Relieving Concerns	Enrichment of the School and Educational Environment	Increasing playgrounds and social activity areas (SA1)<br>Making the school garden suitable for play (SA4)<br>Equipping the classrooms with new toys (SA5)<br>Taking equipment from different sports branches to the school (SA5)<br>Organizing the guidance room in a way that attracts the attention of children (SA5)	5
		Summer Term Planning	Use of schools for various courses in the summer term (SA2)	1

		Uncertainty and Missing Ideas	No other different ideas (SA3)	1
**Practices and Suggestions for Addressing Mothers' Concerns About the Future and Safety**	Pre-Earthquake Concerns	Ensuring School Safety and Communication	Sharing documents regarding the earthquake resistance of the school (SA1)<br>Touring the school building and explaining the renovations made (SA5)<br>Explaining to parents that a safe environment is provided for children (SA4)	3
		Information and Training Activities	Preparation of brochures and training (SA1, SA2)<br>Organizing trainings with experts after the earthquake (SA1)<br>Conducting meetings and private discussions on the measures that can be taken after the crisis (SA3)	4
		Ensuring School Safety and Communication	Sharing documents regarding the earthquake resistance of the school (SA1)<br>Touring the school building and explaining the renovations made (SA5)<br>Explaining to parents that a safe environment is provided for children (SA4)	3
	The Effect of the Earthquake on Concerns	Environmental Regulations	Rapid removal of the remnants of the earthquake (SA1, SA3)<br>Solving housing problems (SA1)<br>Removal of frightening images (SA5)<br>Planning with headman and district national education in landscaping (SA5)	5
		Psychosocial Support and Activities	Preparation of children's and parents' play workshops (SA2)<br>Regular education of parents and students by guidance services (SA5)	2
		Uncertainty and Missing Ideas	No other different ideas (SA3)	1
**Practices and Suggestions for Eliminating Mothers' Concerns About Living Conditions**	What is done to eliminate concerns	Social and Activity-Based Supports	Planning family participation activities (SA1, SA5)<br>Organizing children's festivals in the container city (SA1, SA3)<br>Providing different social environments to children (SA1)<br>Organizing activities to increase peer interaction in different environments (SA4)	6
		Guidance and Institutional Supports	Receiving support from the guidance service (SA5)<br>Directing parents to the relevant institutions for support (SA3)	2
		Training & Courses	Providing full-time training due to the difficulty of staying in the container city (SA2)	1
	Their Views on Relieving Concerns	Rapid Response and Environmental Regulations	Preparing the city for normal functioning (SA1)<br>Creating a quick action plan (SA1)	2

		Guidance and Social Activities	Active use of the guidance service (SA5)<br>Active use of the social activities club at school (SA5)	2
		No idea	No other different ideas (SA2, SA3, SA4)	3
**Practices and Suggestions for Eliminating Mothers' Concerns About Financial Losses and Livelihood Problems**	What is done to eliminate concerns	Coordination and Guidance of Aid	Mediating the support of associations and foundations (SA1, SA4, SA5)<br>Use of philanthropists and ministerial allowances in the procurement of materials (SA5)	4
		Financial Support	Supply of toys and materials for tent and container events (SA1)<br>Meeting the needs of students with aid and allowances (SA1)<br>Distribution of educational materials (SA3)<br>Meeting some of the needs of the children by the school (SA4)<br>Making any financial request (SA1, SA2)	6
	Their Views on Relieving Concerns	Financial Support and Aids	Regular financial assistance (SA1, SA4)<br>Covering education-related transportation, stationery, activity and food expenses with allowances (SA1)<br>Maintaining support and allowances (SA5)	4
		Economic Arrangements and Facilities	Providing tax amnesty to tradesmen, civil servants and workers in the region (SA2)<br>Proper write-off or restructuring of credit card and loan debts (SA2)<br>Minimizing training costs (SA3)	3
**Practices and Suggestions for Eliminating Mothers' Concerns About Psychological Apricots**	What is done to eliminate concerns	Psychological Support and Information	Preparation of family bulletins on coping with the fear of earthquakes (SA1)<br>Organizing seminars on reducing anxiety (SA2, SA5)<br>Providing support by the guidance service (SA3, SA4)	5
	Their Views on Relieving Concerns	Expert Interventions and Psychosocial Support	Providing mass support by specialist physicians in cities where aftershocks continue (SA1)<br>Psychologists and psychiatrists taking an active role in the process through individual and collective trainings (SA1)<br>Regular and sustainable access of psychosocial support teams to earthquake victims (SA2)	3
		Guidance and Family Support Services	Family physicians follow up with families (SA2)<br>Continuation of earthquake-related studies by the guidance service (SA4)<br>egular meetings and seminars between the guidance service and parents (SA5)	3
		Lack of Ideas and Uncertainty	No other different ideas (SA3)	1

**What Needs to Be Done in the Container City to Relieve the Concerns of Mothers**	Vocational Acquisition and Contribution to Production	Opening vocational courses (SA1)<br>Inclusion of citizens in production (SA1)	2
	Normalization and Support	Steps towards the normalization of life (SA2, SA3)<br>Reduction of earthquake reminders (SA2)<br>Transition to prefabricated buildings (SA2)<br>Information based on scientific explanations (SA4)<br>Close follow-up of families (SA5)	6
	Social and Cultural Activities	Increasing social and cultural activities (SA3)<br>Establishment of support offices by various organizations (SA5)	2
**What Needs to Be Done in Terms of Country Policies to Eliminate Mothers' Concerns**	Crisis Management and Rapid Response	Accelerating crisis management (SA1)<br>Demolition and debris removal at the same time (SA1)	2
	Government Intervention and Effective Public Services	Making the presence of the state felt in a concrete way with all kinds of services (SA4)<br>Regular bureaucratic visits (SA5)<br>Listening to problems on site (SA5)	3
	Infrastructure Retrofit	Review of structures according to fault lines after an earthquake (SA2)<br>Reinforcement of buildings (SA2)	2
	International Perspective	Investigation of earthquake experiences and successful practices of other countries (SA3)	1
	Financial Support and Incentives	Maintaining financial and moral support (SA5)<br>Increasing incentives (SA5)	2
